# Development of physiotherapy inherent requirement statements – an Australian experience

**DOI:** 10.1186/1472-6920-13-54

**Published:** 2013-04-16

**Authors:** Andrea Bialocerkowski, Amanda Johnson, Trevor Allan, Kirrilee Phillips

**Affiliations:** 1 Griffith Health Institute, School of Rehabilitation Sciences, Gold Coast Campus, Griffith University, Queensland, 4222, Australia; 2 School of Nursing and Midwifery, University of Western Sydney, Penrith, Australia; 3 Student Equity and Disability Services, University of Western Sydney, Penrith, Australia

**Keywords:** Inherent requirements, Physiotherapy education, Students with a disability, Inclusive curriculum, Inclusive practice

## Abstract

**Background:**

The United Nations Convention on the Rights of People with Disabilities promotes equal rights of people with a disability in all aspects of their life including their education. In Australia, Disability Discrimination legislation underpins this Convention. It mandates that higher education providers must demonstrate that no discrimination has occurred and all reasonable accommodations have been considered and implemented, to facilitate access and inclusion for a student with a disability. The first step to meeting legislative requirements is to provide students with information on the inherent requirements of a course. This paper describes the steps which were taken to develop inherent requirement statements for a 4-year entry-level physiotherapy program at one Australian university.

**Case presentation:**

Inherent requirement statements were developed using an existing framework, which was endorsed and mandated by the University. Items which described inherencies were extracted from Australian physiotherapy professional standards and statutory regulatory requirements, and units contained in the physiotherapy program. Data were integrated into the 8 prescribed domains: ethical behaviour, behavioural stability, legal, communication, cognition, sensory abilities, strength and mobility, and sustainable performance. Statements for each domain were developed using a 5-level framework (introductory statement, description of the inherent requirement, justification for inherency, characteristics of reasonable adjustments and exemplars) and reviewed by a University Review Panel. Refinement of statements continued until no further changes were required. Fifteen physiotherapy inherent requirement statements were developed. The eight domains identified in the existing framework, developed for Nursing, were relevant to the study of physiotherapy.

**Conclusions:**

The inherent requirement statements developed in this study provide a transparent, defensible position on the current requirements of physiotherapy study at one Australian university. These statements are transferable to other physiotherapy programs in Australia due to standardised physiotherapy accreditation requirements. The model and framework could be applied to other health professional courses and used to explore the physiotherapy inherent requirements from an international perspective.

## Background

The United Nations Convention on the Rights of People with Disabilities promotes equal rights of persons with a disability in all aspects of their life, including access to and inclusion in education from the early years through to vocational training and higher education [1]. Although this convention directs the legislative environment in each of the 153 signatory countries, the policies, laws and administrative processes used to secure these rights differ from country and country. From a higher educational perspective, it is important that education providers understand their obligations under the United Nations Convention and specific national legislation so that people with a disability have access to educational opportunities without discrimination and an equal basis with others [1].

In Australia, the Disability Discrimination Act (1992 – as amended) [2] and its subsidiary legislation, the Disability Standards for Education [3], underpins the United Nations Convention of the Rights of People with Disabilities. Within it, disability is defined as 1) total or partial loss of the person’s bodily or mental functions or part of the body; 2) presence in the body of organisms capable or causing disease or illness; 3) malfunction, malformation or disfigurement of a part of a person’s body, 4) a disorder that results in the person learning differently from a person without the disorder; 5) a disorder that affects a person’s thought process, perception of reality, emotions or judgements or that results in disturbed behaviour [2]. This Act [2], together with the Disability Standards for Education [3] and the National Disability Agreement [4], inform higher education providers of the requirements of inclusive education. Inclusive education aims to develop people with disabilities to their full potential so that they can participate effectively in society [1]. This means that education providers must provide curricula to facilitate learning for people with all abilities. Reasonable adjustments to learning experiences are then used to adjust for the effects of a students’ disability to provide equal access to a course [5]. Moreover, Australian legislation binds Australian education providers to take proactive steps to prevent discrimination from occurring [2]. Education providers must be able to demonstrate, through documentary evidence, that no discrimination has occurred, and that all reasonable adjustments have been considered and implemented to facilitate access and inclusion for the student with a disability through their course progression. This requirement may be similar in other countries throughout the world.

There is growing evidence which suggests that people with a disability are pursuing a career in health. In Australia, approximately 11% of 2.2 million 15-64 year olds with a disability are employed in health and community services, and this number is expected to rise into the future [6]. It is, therefore, likely that over the next decades increased numbers of people with a disability will be enrolling in health professional courses. Higher education providers, thus, must consider curriculum design that facilitates inclusive education by addressing barriers to successful higher education for students with a disability, such as physical (e.g. access), social (e.g. negative attitudes) and institutional factors (e.g. non-disclosure, limitations caused by the disability) [7].

### Physiotherapy

Physiotherapy, otherwise known as physical therapy, is a health profession which is “concerned with identifying and maximising quality of life and functional movement potential, within the spheres of promotion, prevention, maintenance, intervention/treatment, habilitation and rehabilitation. This encompasses physical, psychological, emotional and social well-being” [8]. The education of entry-level physiotherapy students internationally is underpinned by the World Confederation for Physical Therapy (WCPT) guidelines, which state that curriculum must qualify the physiotherapist for practice as an independent, autonomous professional, and incorporate region-specific professional and statutory regulatory body requirements [9]. In Australia, these requirements include:

• Mandatory requirements by Law - the Government of Queensland Health Practitioner Law Act [10]

• Entry-level physiotherapy competency standards – Australian Standards for Physiotherapy [11]

• Australian codes of physiotherapy practice – Australian Physiotherapy Association Code of Conduct [12], Code of Conduct for Registered Health Practitioners [13].

The WCPT policy on Physical Therapist Professional Entry-level Education lists characteristics inherent in a practicing physiotherapist, which also should be incorporated into curriculum design (e.g. assessment, diagnosis, prognosis, intervention, re-assessment, communication, critical evaluation, evidence based practice, research) [9]. However no policies have been developed to guide educators on how to make reasonable adjustments to facilitate students with a disability to meet these requirements.

### Students with a disability and physiotherapy

A small body of evidence exists on students with a disability studying physiotherapy. Ward et al [14] interviewed 43 physical therapists and 6 physical therapy assistants with a disability to describe their education experiences. Ninety-one percent of participants reported the onset of their disability was prior to admission. Diagnoses included disorders that were visible (e.g. amputation, stroke, cerebral palsy) and non-visible (e.g. chronic fatigue syndrome, asthma). Sixty-two percent of participants reported that they required adjustment in academic and or practice placement settings. Rangel et al [15] found that 77% of 112 US physiotherapy schools educated students with learning disabilities. Fifty-one percent of these schools reported that some students had back injuries. Other disabilities reported included auditory and visual disorders, mental health issues, speech impediments, and orthopaedic, neurological and cardiorespiratory disorders. Sharby and Roush [16] reported an increase in new graduate physiotherapists requesting disability-related adjustments on the US licensure examination from 1 to 4% from 2001 to 2005. The most frequently reported diagnoses were psychiatric, learning difficulties and attention deficit hyperactivity disorder.

Epidemiological data is not available on the prevalence of Australian students with a disability studying physiotherapy, as it has not been systematically collected from Australian physiotherapy programs. Anecdotal evidence suggests that lack of disclosure by students makes it difficult to quantify the extent of the issue. Lack of disclosure has been cited as a major issue in the Higher Education inclusion literature [7]. Anecdotally, however, there is similarity in the types of disorders present in US and Australian physiotherapy student cohorts.

Evidence suggests that reasonable adjustments can be made to allow students to participate in and complete physiotherapy education programs, once disclosure has occurred, without compromising academic and professional standards, and legal requirements [14,17-19]. However there is a paucity of published evidence on how decisions on reasonable adjustments are made for physiotherapy students, and the impact of these on learning and student progression.

### Inherent requirements

Inherent requirements of study in a course are considered as the cornerstone of inclusive education [20]. The term “inherent requirement” does not have an internationally-accepted definition. Therefore at the University of Western Sydney (UWS), it was defined, in the local context, as

“the fundamental components of a course or unit, that are essential to demonstrate the capabilities, knowledge and skills to achieve the core learning outcomes of the course or unit, while preserving the academic integrity of the university’s learning, assessment and accreditation process. *Note*: *making a requirement compulsory does not necessarily make it an inherent requirement*” [21].

This definition takes into account the balance between the legislative requirement of inclusiveness and attainment of professional standards. Higher education providers are not mandated to provide inherent requirement statements for courses on offer, either in Australia or internationally. However, it has been recommended that inherent requirement statements providing a shared understanding of the inherency within a course of study and should be used to assist:

1. Future students to make informed decisions about the study choices, in particular their ability to meet the challenges of study in programs which contain practice placements (e.g. physiotherapy) before accepting a university offer [19,21,22]

2. Continuing students to make informed decisions regarding their capacity to continue in their program of study [21,22]

3. Disability services and academics staff members to develop and document reasonable adjustments for students with a disability, which meet legislative, professional and academic requirements [19,21,22].

Rankin et al state that well informed students understand how disclosure impacts positively on the success of their study [19]. Policies are therefore required to clearly outline the conditions under which students with different abilities can participate in and complete programs of study.

### Inherent requirements of physiotherapy study

**Figure 1:**
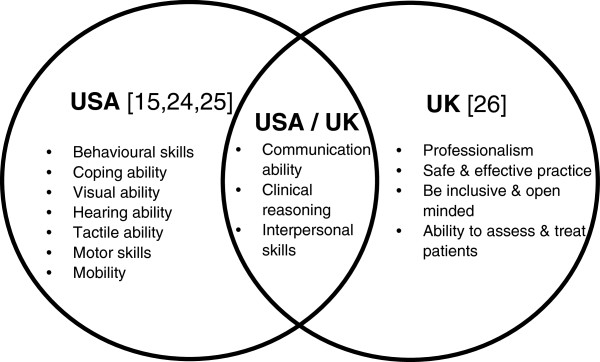
Essential physiotherapy student functions, in the USA and the UK.

In Australia, there is a paucity of information on the requirements of physiotherapy study and possible reasonable adjustments for students with a disability. Our systematic web-based search of Australian physiotherapy programs found that one third of Australian universities provided generic information on the requirements of physiotherapy study. This information, however, offers little guidance to students with a disability. Information on requirements of physiotherapy study tended to focus on “fitness to practice”. However, by definition, “fitness to practice” encompasses clinical competence, acceptable professional behaviour and freedom from impairment [23]. This is at odds with inclusive education, which makes learning accessible irrespective of impairment or disability [5]. The international literature also offers little guidance, as there is a lack of agreement between the fundamental skills required as a physiotherapy student in the USA [15,24,25] and the UK [26] (Figure 1). Moreover, the scope of physiotherapy practice, the healthcare funding and systems, and cultural factors differ between Australia and other developed countries, such as the USA and the UK [27].

This paper addresses this gap, by describing the steps which were taken to develop inherent requirement statements for a 4-year entry-level physiotherapy program at one Australian university, the University of Western Sydney (UWS), so that clear and transparent information could be provided to future and continuing physiotherapy students, disability services and academic staff members regarding the requirements of physiotherapy study.

### Case presentation

#### Context

Inherent requirement statements were developed for the 4-year entry level physiotherapy program at UWS, Australia. This program consisted of two seamless combined degrees: Bachelor of Health Science and Master of Physiotherapy, and was commenced in 2010. The physiotherapy program is located in the School of Science and Health, which offers other health professional education programs with the same degree structure (occupational therapy, podiatric medicine, traditional Chinese medicine) [28]. The vision of the physiotherapy program is to attract students who reside in Greater Western Sydney to study and then to practice physiotherapy in the region upon graduation. This vision supports the rapid expansion of the population in Greater Western Sydney and its need for healthcare services [29].

## Materials

**Figure 2:**
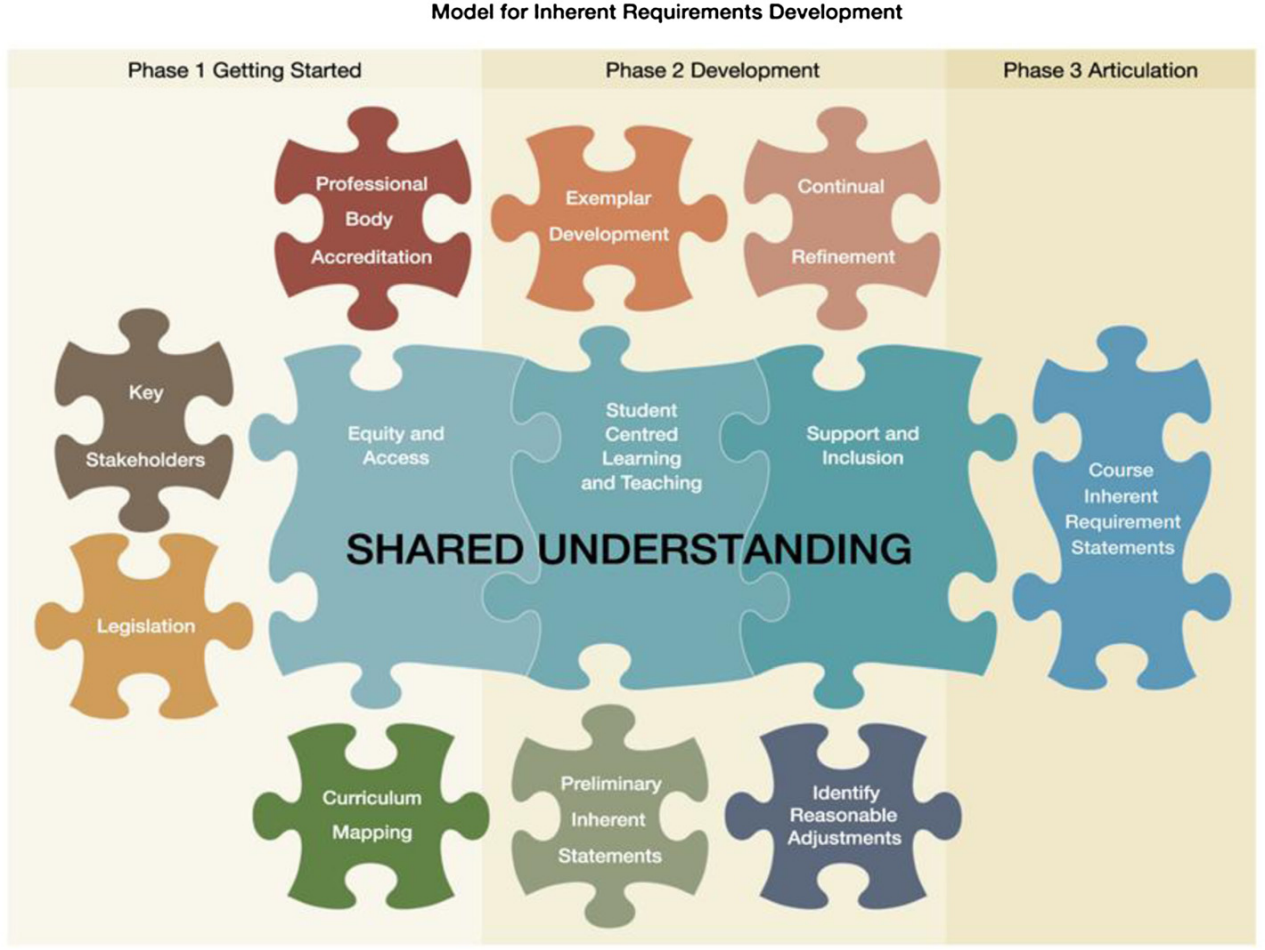
**Model for inherent requirements development [**[22]**].**

The inherent requirement model and 5-level framework, which was developed during the Inherent Requirements of Nursing (IRONE) project, was used to develop physiotherapy inherent requirement statements [21,22,30]. This model and framework was endorsed by the UWS Academic Senate in 2011, where it was mandated that inherent requirement statements must be developed (using the IRONE model and framework) for all courses by 2013. This well aligns to the University’s mission of providing a supportive learning environment so that students can achieve their full potential within their chosen field [31]. Moreover, the IRONE model and framework offer advantages over published models of inclusion, such as the PracAbility Framework [32] as it explicitly defines how inherent requirements should be developed. As shown in Figure 2, the inherent requirement development framework consists of three phases: 1) Getting Started, 2) Development, and 3) Articulation. This paper details the development of the physiotherapy inherent requirement statements (Phases 1 and 2) and discusses the progress made in phase 3 (Articulation).

### Stakeholders

A diverse group of stakeholders participated in development of the physiotherapy inherent requirement statements. These stakeholders included all UWS physiotherapy academic staff members, who collectively had expertise spanning curriculum development, disability and clinical practice placements. This provided a comprehensive and holistic approach to inherent requirement statement development [22]. Moreover, engagement with all UWS physiotherapy staff provided the opportunity of “ownership” of the inherent requirement statements by the staff collective.

In addition, a University inherent requirement reference group oversaw this project. The reference group consisted of the IRONE project leader (a nursing academic with extensive experience in curriculum); Head of the University of Western Sydney Disability Services and the IRONE Project Officer. The reference group provided independent feedback to the inherent requirement statement developers to ensure that there was congruence with the IRONE framework and a shared understanding of inherency across courses at UWS.

## Methods

**Phase 1**, Getting Started, was underpinned by the recommendations by WCPT [9] and the Australian Human Rights Commission [33] to include professional and statutory regulatory body requirements and the UWS physiotherapy curriculum to inform inherent requirement statement development. The data sources included:

1. Mandatory requirements by Law, which were gained from the Government of Queensland Health Practitioner Regulation National Law Act [10]

2. Entry level physiotherapy competency standards, which were gained from the Australian Standards for Physiotherapy [11]. These standards for the basis of accreditation of Australian physiotherapy programs

3. The codes of physiotherapy practice in Australia, which were gained from the Australian Physiotherapy Association Code of Conduct [12] and the Code of Conduct for Registered Health Practitioners [13]

4. The University of Western Sydney (UWS) Bachelor of Health Science/Master of Physiotherapy curriculum map which consisted of the learning outcomes of 32 teaching units.

**Phase 2**, Development, involved extraction of inherent requirement items from the four data sources by one experienced physiotherapy academic. Inherent requirements were defined as fundamental / core tasks required by a student undertaking a physiotherapy course [21,22]. Mandatory requirements by Law were extracted for registration as a physiotherapist as well as for student registration. Physiotherapist registration requirements were examined closely for relevance to physiotherapy students and the inherency of studying physiotherapy. The Standards for Physiotherapy [11] and codes of physiotherapy practice in Australia [12,13] were deconstructed, as these standards provide information on the knowledge, skills, attitudes and behaviours of entry level physiotherapists, not physiotherapy students. Deconstruction allowed the identification of the underlying requirements which were inherent, and enabled physiotherapy knowledge, skills and attitudes to be built on throughout the physiotherapy course.

Each extracted item was subsequently scrutinised to ensure it represented an inherent requirement; that is a core activity that is considered essential in the study of physiotherapy [21,22]. Specifically, items which focused on the outcome of the core activity were retained as inherent requirements. In contrast, those items which represented how activities were performed were not considered inherent requirements and were removed from the list, as they focused on the process associated with an activity [33]. These preliminary inherent requirements were then discussed among UWS physiotherapy academic staff to gain their perspective on the comprehensiveness of the list. This process involved educating the physiotherapy academic staff members on the definition of an inherent requirement and gaining, first, their feedback and then their consensus on the preliminary list of inherent requirements.

The preliminary list generated was then integrated into the IRONE inherent requirement framework, which consists of eight domains: 1) ethical behaviour, 2) behavioural stability, 3) legal, 4) communication, 5) cognition, 6) sensory abilities, 7) strength and mobility, and 8) sustainable performance [22]. Four of the eight domains (communication, cognition, sensory abilities, strength and mobility) had several sub-domains (Table 1). Each domain consisted of five levels:

• Level 1: Introductory statement

• Level 2: Description of the inherent requirement

• Level 3: Justification of the inherency

• Level 4: Characteristics of reasonable adjustments

• Level 5: Exemplars both from classroom and clinical settings.

**Table 1:** **Inherent requirement framework: categories and sub-categories**[22]

	**Category**	**Sub-category**
1.	Ethical behaviour	
2.	Behavioural stability	
3.	Legal	
4.	Communication	• Verbal
		• Non-verbal
		• Written
5.	Cognition	• Knowledge and cognitive skills
		• Literature (language)
		• Numeracy
6.	Sensory abilities	• Visual
		• Auditory
		• Tactile
7.	Strength and mobility	• Gross motor skills
		• Fine motor skills
8.	Sustainable performance	

The draft physiotherapy inherent requirement statements were then reviewed by the University Inherent Requirement Reference Group. Critique, ongoing dialogue and subsequent refinement of the physiotherapy inherent requirement statements continued until no further changes were identified by the Reference Group or physiotherapy academic staff [22]. A final version of the inherent requirement statements was reviewed by the UWS physiotherapy academic staff, to ensure that the exemplars reflected the scope of physiotherapy practice in Australia, and covered both academic and clinically-focused activities.

### Physiotherapy inherent requirement statements

Fifteen physiotherapy inherent requirement statements were developed and are found, in full, at http://www.uws.edu.au/ir/inherent_requirements/inherent_requirements_for_physiotherapy_courses[34]. The inherent requirement statements covered all eight IRONE domains: ethical behaviour, behavioural stability, legal, communication, cognition, sensory abilities, strength and mobility and sustainable performance (Table 2)[22]. More than one inherent requirement statement was developed for four domains:

• Communication: statements were developed for verbal, non-verbal and written communication

• Cognition: statements were developed for knowledge and cognitive skills, numeracy and literacy

• Sensory abilities: statements were developed for visual, auditory and tactile sensory abilities

• Strength and mobility: statements were developed for gross motor and fine motor skills.

**Table 2:** Justification of the physiotherapy inherent requirement domains, based on legislation, physiotherapy standards and codes of conduct

**Inherent requirement domains [**[21]**]**	**Mandatory requirements by law [**[10]**]**	**Entry level physiotherapy competency standards [**[11]**]**	**Australian physiotherapy association code of physiotherapy practice [**[12]**]**	**Code of conduct for registered health practitioners [**[13]**]**
Ethical behaviour		Standard 1: Ethical behaviour	Principle 1: APA members must respect the rights, needs and dignity of all individuals	Standard 1: Providing good care
		*1.1 Demonstrate practice that is ethical and in accordance with relevant legal and regulatory requirements*		Standard 2: Working with patients or clients
			Principle 5: APA members must respect the confidentiality, privacy and security of client health information	
				Standard 7: Professional behaviour
		Standard 7: Implement safe and effective physiotherapy interventions		
			Principle 7: APA members must act in a manner which maintains the good standing of the physiotherapy profession	
		*7.1 Obtain informed consent for the intervention*		
		Standard 9: Operate efficiently across range of settings		
		*9.4 Operate within own role and according to responsibilities*		
Behavioural stability		Standard 9: Operating across a range of settings	Principle 3: APA members must practice in a safe, competent and accountable manner	Standard 1: Providing good care
		*9.2 Work effectively within a team*		
		*9.3 Manage own work schedule to maximise safety, efficiency and effectiveness*		
		*9.4 Operate within own role and according to responsibilities*		
Legal	English language proficiency	Standard 1: Ethical behaviour	Principle 2: APA members must comply with laws and regulations governing the practice of physiotherapy in Australia	Standard 2: Working with patients or clients
	Criminal history - in good standing	*1.1 Demonstrate practice that is ethical and in accordance with relevant legal and regulatory requirements*		
				Standard 5: Minimising risk
			Principle 5: APA members must respect the confidentiality, privacy and security of client health information	
			Principle 9: APA members must comply with the Constitution and Regulations of the Australian Physiotherapy Association	
		Standard 2: Communicate effectively		
		*2.5 Prepare and provide documentation according to legal requirements and accepted procedures and standards*		
Communication (verbal, non-verbal, written)	English language proficiency	Standard 2: Communicate effectively	Principle 1: APA members must respect the rights, needs and dignity of all individuals	Standard 1: Providing good care
		*2.1 Communicate effectively with the client*		Standard 2: Working with patients or clients
			Principle 3: APA members must practice in a safe, competent and accountable manner	
		*2.2 Adapt communication style recognising cultural safety, and cultural and linguistic diversity*		Standard 3: Working with other practitioners
				Standard 7: Professional behaviour
		*2.3 Communicate effectively with other service providers*	Principle 6: APA members must communicate and co-operate with colleagues and relevant agencies in the best interests of their clients and the wider community	
		*2.4 Prepare and deliver presentations*		
		*2.5 Prepare and provide documentation according to legal requirements and accepted procedures and standards*		
		Standard 6: Develop a physiotherapy intervention plan		
		*6.2 Set realistic short and long term goals with the client*		
		*6.5 Prioritise intervention plan in collaboration with the client*		
		Standard 7: Implement safe and effective physiotherapy interventions		
		*7.1 Obtain informed consent for the intervention*		
Cognition (knowledge and cognitive skills, literacy, numeracy)	Professional development	Standard 3: Assess, interpret and apply information to continuously improve practice	Principle 1: APA members must respect the rights, needs and dignity of all individuals	Standard 1: Providing good care
				Standard 4: Working within the healthcare system
				Standard 6: Maintain professional performance
		*3.1 Demonstrate a working knowledge and understanding of theoretical concepts and principles relevant to physiotherapy practice*	Principle 3: APA members must practice in a safe, competent and accountable manner	
			Principle 4: APA members must strive for standards of excellence in physiotherapy	
		*3.2 Apply contemporary forms of information management to relevant areas of practice*		
		*3.3 Apply and evidence-based approach to won practice*		
				Standard 10: Undertaking research
		*3.4 Acquire and apply new knowledge to continuously improve own practice*		
		Standard 4: Assess the client		
		*4.1 Collect client information*	Principle 8: APA members must strive to contribute to the development and implementation of health service delivery which enhances the health status of the community and promotes social justice	
		*4.2 Form a preliminary hypothesis*		
		*4.3 Design and conduct an assessment*		
		Standard 5: Interpret and analyse the assessment findings		
		*5.1 Compare findings with ‘normal’*		
		*5.2 Compare findings with what is expected for the condition*		
		*5.3 Prioritise client’s needs*		
		*5.4 Re-evaluate as required*		
		*5.5 Identify areas that are outside skills and expertise and refer client appropriately*		
		Standard 6: Develop a physiotherapy intervention plan		
		*6.1 Develop rationale for physiotherapy intervention*		
		*6.2 Set realistic short and long term goals with client*		
		*6.3 Select appropriate intervention*		
		*6.6 Determine plan of evaluation that uses valid and reliable outcome measures*		
		Standard 8: Evaluate the effectiveness and efficiency of physiotherapy interventions		
		*8.1 Monitor the outcomes of the intervention*		
		*8.2 Evaluate the outcomes of the intervention*		
		*8.3 Determine the modifications to intervention*		

Sensory abilities (visual, auditory, tactile)		Standard 2: Communicate effectively	Principle 3: APA members must practice in a safe, competent and accountable manner	Standard 1: Providing good care
		*2.1 Communicate effectively with client*		
		*2.2 Adapt communication style recognising cultural safely, and cultural and linguistic diversity*		
		*2.3 Communicate effectively with other service providers*		
		*2.4 Prepare and deliver presentations to groups*		
		*Prepare and provide documentation according to legal requirements and accepted procedures and standards*		
		Standard 4: Assess the client		
		*4.1 Collect client information*		
		*4.3 Design and conduct an assessment*		
		*4.4 Conduct assessment safely*		
		Standard 7: Implement safe and effective physiotherapy interventions		
		*7.3 Implement intervention safely and effectively*		
		Standard 8: Evaluate the effectiveness and efficiency of physiotherapy interventions		
		*8.1 Monitor the outcomes of intervention*		
Strength and mobility (gross motor, fine motor)		Standard 4: Assess the client	Principle 3: APA members must practice in a safe, competent and accountable manner	Standard 1: providing good care
		*4.3 Design and conduct an assessment*		
		*4.4 Conduct assessment safely*		
		Standard 7: Implement safe and		
		effective physiotherapy interventions		
		*7.3 Implement intervention safely and effectively*		
		Standard 8: Evaluate the effectiveness and efficiency of physiotherapy interventions		
		*8.2 Evaluate the outcomes of the intervention*		
Sustainable performance		Standard 9: Operate across a range of settings	Principle 3: APA members must practice in a safe, competent and accountable manner	Standard 8 : Ensuring practitioner health
		*9.1 Manage own work schedule to maximise safety, efficiency and effectiveness*		
		*9.4 Operate within own role and according to response*		

As per the IRONE framework, all physiotherapy inherent requirement statements consisted of five levels:

1. An introductory statement to communicate the intent. This statement is underpinned by professional and statutory regulatory requirements [9,33]

2. A description of the inherent requirement, which provides information on the expectation of knowledge and behaviour of physiotherapy students

3. Justification of the inherency, including the impact on the client and the student

4. Characteristics of reasonable adjustments

5. Examplars from both classroom and clinical settings, as anecdotal evidence suggests that students often consider these education settings as distinct, with different policies and expectations governing them. Examplars create a shared understanding among those involve in using the inherent requirement statements – the student with a disability, the disability advisor and the academic staff member.

Table 3 provides an example (in full) of one of the physiotherapy inherent requirement statements – ethical behaviour.

**Table 3:** **Physiotherapy inherent requirements – ethical behaviour**[34]

1.	Introduction	Physiotherapy is a profession that is governed by code of conduct and standards where physiotherapists are both accountable and responsible for ensuring safe and professional behaviour in all contexts
2.	Description:	Demonstrates knowledge and engages in ethical behaviour
3.	Justification of inherent requirement:	• Compliance with the standards and codes facilities safe, competent interactions and relationships with people to ensure that their physical, psychological, emotional and spiritual wellbeing is not placed at risk• Compliance with the standards and codes facilities safe, competent interactions and relationships with people to ensure that the student’s physical, psychological, emotional and spiritual wellbeing is not placed at risk
4.	Adjustments	Adjustments must not compromise codes of conduct or result in unethical behaviour
5.	Exemplars:	• Complying with academic and non-academic misconduct policies in both academic and clinical settings• Complying with medico-legal requirements related to informed consent, privacy, confidentiality with client information in academic and clinical settings

## Discussion

This paper describes the steps which were taken to develop inherent requirement statements for a 4-year entry level physiotherapy program at one Australian university, using an established inherent requirement model and framework, which is underpinned by recommendations by WCPT [9] and the Australian Human Rights Commission [33]. Using Australian-specific physiotherapy professional and statutory regulatory requirements as data sources means that the statements provide a robust, defensible position on the current requirements of study at UWS. Their use will meet the University’s obligations under Australian legislation [2] and the United Nations convention on the Rights of people with a Disability [1] to be proactive in preventing discrimination against students with a disability as well as facilitating access to the physiotherapy course for students with a disability.

The UWS physiotherapy inherent requirement statements substantially add to the body of knowledge in this area. The data sources used in this study mirror those which have been recommended to inform inherent requirement development [9,33]. This range of data sources has not been previously used in published physiotherapy studies. As expert opinion was the solely used to establish physiotherapy student requirements in the USA and the UK [15,24-26,35], respondent bias was likely to have influence previous results. Respondent bias was reduced in this study by using of a range of accepted data sources and by oversight by an independent University inherent requirement reference group [25]. Moreover, the use of the IRONE framework meant that the inherency was explicitly articulated, through rich description of the inherency plus exemplars, and characteristics for reasonable adjustments provided [22]. This is in contrast to other literature in this area, which merely listed essential functions or physiotherapy student skills [15,25,26,35].

Fifteen inherent requirements statements were developed for the study of physiotherapy at UWS [34]. These statements covered the same domains as those identified in the Bachelor of Nursing IRONE project [22]. This provides evidence of the content validity of these eight domains: 1) ethical behaviour, 2) behavioural stability, 3) legal, 4) communication, 5) cognition, 6) sensory abilities, 7) strength and mobility, and 8) sustainable performance [22]. Further development of inherent requirement statements for other health courses may support inherencies which are common across courses which educate health professionals. This process could be facilitated by use of the IRONE development model and framework [21,22].

Differences exist between the inherent requirements to study physiotherapy at UWS and the essential skills required by US and UK physiotherapy students [15,26] (Table 4). For example, in the USA and the UK, legal requirements of physiotherapy study were not identified as essential. In Australia, legal requirements of the study and practice of physiotherapy are mandated by the Health Practitioner Regulation Law Act [10]. Universities are required to register all physiotherapy students with the Physiotherapy Board of Australia, and students must abide by legislative requirements, such as mandatory reporting of “notifiable conduct” of substandard practice or conduct, or serious impairment of students and practitioners. If students cannot meet these legal obligations (for example having good standing with respect to criminal history), their ability to complete the physiotherapy course may be affected. Prospective students, therefore, should be informed of the legal requirements of physiotherapy study, including student registration. This would enable them to undertake a self-assessment of their suitability to meet the inherency of physiotherapy study.

**Table 4:** Comparison of the UWS physiotherapy inherent requirements with the US and UK essential functions for physiotherapy

**Current study**	**USA [**[15]**,**[24]**,**[25]**]**	**UK [**[26]**]**
Ethical behaviour		Behave in a professional manner
		Practice safely and efficiently
		Be inclusive and open minded
Behavioural stability	Behavioural skills	
	Coping skills	
Legal		
Communication (verbal, non-verbal, written)	Communication ability	General communication skills
	Interpersonal skills	Interpersonal skills
		Listening skills
		Team work skills
		Verbal communication skills and fluency
Cognition (knowledge and cognitive skills, literacy, numeracy)	Critical thinking	Clinical reasoning
		Integration and synthesis of information
Sensory abilities (visual, auditory, tactile)	Visual ability	Be able to assess, examine and treat patients
	Hearing ability	
	Tactile ability	
Strength and mobility (gross motor, fine motor)	Motor skills	Be able to assess, examine and treat patients
	Mobility	
Sustainable performance		

Sustainable performance has not been previously identified as an important factor in physiotherapy study. Sustainable performance was identified in the IRONE project as sufficient physical and mental endurance that is required to perform multiple tasks over an assigned period of time, to provide safe and effective care [22]. This inherent requirement mirrors what is required for contemporary clinical practice in Australia, as defined by the Australian Standards for Physiotherapy [11], the Australian Physiotherapy Association Code of Conduct [12] and the Code of Conduct for Registered Health Practitioners [13] (Table 3). Without adequate sustainable performance students tend to experience difficulty within both the university and clinical education settings. This may result in putting the students, the public and the university at risk.

The IRONE developmental model [22] was explicitly followed to develop the physiotherapy inherent requirement statements. This model was simple to understand and operationalise, yet generic in nature. This would allow other courses at universities in both Australia and internationally to follow this process. The range of data sources used in this project (legislation, professional competency standards, professional codes of conduct and curriculum) are available for physiotherapy in many countries around the world (Table 5) and for other health courses such as medicine, nursing and the allied health processions. However, it is acknowledged that the IRONE model and framework has not been vigorously testing. However, its eight domains cover the characteristics inherent in a practising physiotherapist, as defined by WCPT [9], as well as documented behaviours that question the competence of physiotherapy students [36]. Evaluation and possible subsequent adaptation of the model and framework, as well as the physiotherapy inherent requirement statements, may be required once the statements have been in regular use. In addition, the physiotherapy inherent requirement statements will require updating if / when physiotherapy professional and or statutory regulatory requirements change.

**Table 5:** Examples of data sources for future development of physiotherapy inherent requirement statements– an international perspective

**Country**	**Legislation**	**Standards of physiotherapy**	**Codes of physiotherapy conduct**
Canada	Canadian Human Rights Act [38]	Essential competency profile for physiotherapists in Canada [39]	Code of ethics and rules of conduct [40]
Great Britain	Equality Act 2010 [41], Special Education Needs and Disability Act [42]	Core standards of physiotherapy practice [43]	Code of professional values and behaviour [44], professional rules [45]
New Zealand	New Zealand Public Health and Disability Act [46]	Standards of physiotherapy practice [47]	Aotearoa New Zealand physiotherapy code of ethics and professional conduct [48]
USA	Americans with Disability Act Amendment Act [49]	Criteria for standards of practice for physical therapy [50]	Code of ethics for the physical therapist [51]

Articulation, Phase 3 of the model (Figure 2), is currently underway. The method of articulation of these statements has been dictated by the University, but it is well aligned with the findings by Rangel et al, who found that the majority of US physiotherapy schools made students aware of essential functions before or at admission [15]. Moreover, Ward et al found that most students in a US physiotherapy program reported that their disability occurred prior to their admission into the program [14]. At UWS, the physiotherapy inherent requirement statements have been publically available since April 2012 [34]. Future students can access this information from course websites and from the University handbook. Inherent requirement statements also form part of the admission process. Students cannot proceed without first hitting the relevant course link before signing off on their letter of offer. These mechanisms support potential and continuing students to make informed decisions about their course of study. When future students make inquiries regarding the study of physiotherapy at the University of Western Sydney, they are directed to the physiotherapy inherent requirement webpage and the handbook, which are linked electronically. Providing future students with information regarding the inherency of studying physiotherapy has initiated discussion on reasonable adjustments, at a time when students are making decisions regarding their course preferences. Current physiotherapy students have also been alerted to the inherent requirement statements.

## Conclusions

To increase the accessibility of physiotherapy study to people with a disability, a process of using inherent requirement statements must be in place for universities to promote inclusion, manage risk and ensure compliance with legislation. The inherent requirement statements described in this paper provide a robust, defensible position on the current requirements of physiotherapy study at one Australian university [34]. These statements are immediately transferable to other physiotherapy programs in Australia because of the standardised physiotherapy accreditation requirements [37]. Although physiotherapy legal requirements and professional standards, disability legislation and physiotherapy curricula differ between countries [27], the IRONE development model and framework [22] utilised in this study could be used in the future to explore the inherent requirements of physiotherapy study from an international perspective (Table 5), and could also be applied to other health professional courses.

## Abbreviations

UK: United Kingdom; US: United States (of America); UWS: University of Western Sydney; WCPT: World Confederation for Physical Therapists.

## Competing interests

The authors declare they have no competing interests.

## Authors’ contribution

AB: substantial contributions to conception, design and acquisition of data, analysis and interpretation of data, drafted the manuscript and revised it critically for intellectual content, gave approval of this version of the manuscript. AJ: Substantial contributions to the conception and design of the project, and interpretation of the data, critically revised the manuscript for intellectual content, gave approval of this version of the manuscript. TA: Substantial contributions to the conception and design of the project, and interpretation of the data, critically revised the manuscript for intellectual content, gave approval of this version of the manuscript. KP: Substantial contributions to the conception and design of the project, and interpretation of the data, critically revised the manuscript for intellectual content, gave approval of this version of the manuscript.

## Pre-publication history

The pre-publication history for this paper can be accessed here:

http://www.biomedcentral.com/1472-6920/13/54/prepub
